# The Applicability of the Theory of Planned Behavior for Research and Care of Female Genital Cutting

**DOI:** 10.1007/s10508-020-01716-9

**Published:** 2020-04-26

**Authors:** R. Elise B. Johansen

**Affiliations:** Norwegian Centre for Violence and Traumatic Stress Studies (NKVTS), PB 181, 0409 Nydalen, Oslo, Norway

## Setting the Scene

I strongly appreciated being invited to write a Commentary on Brady, Connor, Chaisson, Mohamed, and Robinson’s (2019) Target Article as I found the paper to be a breath of fresh air into research on female genital cutting (FGC). I particularly appreciated Brady et al.’s efforts to formulate a tool that supports providers in offering improved health care for girls and women subjected to infibulation and provides a useful theoretical framework and simple model that encompasses the major complexities regarding FGC decision making.

Brady et al.’s (2019) Target Article offers a much needed broadening of perspective from the more one-dimensional theory of FGC as a social convention that has dominated research and activism over the last decades (Johansen, 2019; Johansen, Diop, Laverack, & Leye, 2013; Mackie, 2000; UNICEF, 2005, 2013). The theory of FGC as a social convention contributed significantly to our understanding of the social aspects of FGC. This theory, however, risks portraying individuals and families as totally determined by sociocultural context. And while it can be used to understand how a change in social norms can lead to fundamental change in behavior, it is not well suited to explore the initial stages of change or individuals and groups who do not follow social norms (Johansen, 2017c).

Furthermore, I find the model of planned behavior particularly suited to today’s contexts of changing FGC discourses and practices, which takes place both in FGC practicing countries and in places where the practice mainly concerns migrants from FGC practicing countries. It seems particularly pertinent to apply the theory of planned behavior (TPB) to the issue raised in the Target Article, that of deinfibulation in diaspora.

In a contemporary context, where different perceptions and views of FGC, infibulation, and deinfibulation coexist, the application of this model for research and care purposes must be based on a broader knowledge of the topic. Outlining some of these nuances is my main contribution in this Commentary.

My comments are based on experience from more than 20 years of working on issue related to FGC, in research, policy development, and interventions. In a sense, the model provided by Brady et al. (2019) answers a concern I outlined 16 years ago with the rhetorical title “Just a Snip?” (Johansen, Barre, Sundby, & Vangen, 2004), highlighting how the providers’ perception of deinfibulation as a minor and uncomplicated surgery failed to take into account how profound and complex a decision it was for those affected. Since then, services have improved significantly (Johansen, 2017c; Johansen, Ziyada, Shell-Duncan, Kaplan, & Leye, 2018). However, we still find a huge resistance to deinfibulation, low usage of services, as well as cases of insufficient culturally sensitivity from certain providers (Johansen, 2019; Johansen & Ahmed, 2020; Ziyada, Lien, & Johansen, in press). Thus, I think the model provided by Brady et al. can be a useful tool to bridge the gap between the way in which infibulation and deinfibulation is perceived by patient and provider.

My Commentary will thus partly be formulated as a test of the applicability of Brady et al.’s (2019) model on my empirical experiences in the field. I will also highlight the need to complement the model with insights provided by major nuances and variations in the field (Gele, Johansen, & Sundby, 2012; Johansen, 2002, 2004, 2006, 2007, 2017a, b; 2019; Johansen et al., 2013, 2018; Vangen, Stoltenberg, Johansen, Sundby, & Stray‐Pedersen, 2002). I will discuss these within the framework of each of the main categories in the theory: attitude, perceived norms, and perceived control, as well as their interconnectedness. Furthermore, I will further argue for the importance of addressing “the elephant in the room,” the cultural meaning underpinning all these factors, namely perceptions of female sexuality. The cultural meaning of infibulation, and the resistance to deinfibulation, is intimately intertwined with ideas of female sexuality. I will complement this introduction by delineating the traditional cultural meaning of infibulation.

## Infibulation as a Cultural Practice

Infibulation, the most severe of the four types of FGC, is most common among ethnic Somalis across the countries in which they have traditional settlements (Somalia, including Somaliland and Puntland, Djibouti, Ethiopia, and Kenya), among Sudanese, and among Eritreans (UNICEF, 2013). The seal of skin covering the clitoris, urethra, and part of the vaginal opening that is created through infibulation is commonly understood as necessary to create, protect, and prove virginity (Johansen, 2017a). Thus, its value is intrinsically intertwined with perceptions of female sexuality and, through this, morality. Traditionally, only an infibulated woman would be considered a virgin, and thus, a proper and reliable woman is eligible for marriage and adulthood. In contrast, an uninfibulated woman is perceived as immoral and subjected to severe negative social sanctions. This is perceived as making her unfit for marriage and motherhood, the ultimate measure of maturity, respectability, and success as a woman (Ziyada et al., in press). However, some form of deinfibulation is also necessary to enable sexual intercourse and childbirth and thus of fundamental importance for cultural values related to marriage and motherhood (Johansen, 2007). Thus, both infibulation and deinfibulation are intimately linked with core cultural values related to women’s sexuality, fertility, and respectability.

In diaspora, some of these traditional values are changing. Several studies have found increasingly negative attitudes to infibulation (Gele et al., 2012; Johansen, 2017a, 2019; Johnsdotter & Essèn, 2016; Mestre i Mestre & Johnsdotter, 2019; Vogt, Efferson, & Fehr, 2017; Wahlberg, Johnsdotter, Selling, Källestål, & Essén 2017) and some acceptance of surgical deinfibulation (Tvenge & Andersen, 2017). Nevertheless, there remains substantial resistance to deinfibulation. Many women go on living with severe health problems that could have been significantly improved by surgical deinfibulation (Ziyada et al., in press). This resistance is mainly explained by a high degree of continuity in underlying values tying infibulation to sexuality. This includes the protection and evidence of premarital virginity as outlined previously, as well as a measure to secure male sexual pleasure and, hence, marital stability (Alhassan, Barrett, Brown, Kwah & Reisel, 2016; Berg & Denison, 2013a, b; Johansen, 2017a, b, 2018; Ziyada et al., in press). Thus, the guidance offered in Brady et al.’s (2019) Target Article could be a useful tool in approaching these concerns.

## Some Strength of the Theory of Planned Behavior for Understanding Female Genital Cutting Decision Making

The theory of planned behavior builds on evidence indicating that declared intentions are a better predictor for behavior than attitude. However, this can depend on the situation. In a country where FGC is illegal and intention to subject someone to FGC could cause legal repercussions, attitude–especially if assessed indirectly (Vogt et al., 2017)–might be more reliable than intention. However, when assessing deinfibulation in diaspora, intention might be more reliable, as such intentions would not challenge the law. Thus, the applicability of the theory is likely to vary with the cultural, moral, and legal sensitivity to the aspect of FGC studied.

As such, the theory of planned behavior combines elements from both theories of individual behavioral change and the theory of FGC as a social convention (Mackie, 2000). The theory of FGC as a social convention claims that once FGC has become a social convention, it is locked into place, and no individual or family member can abandon the practice due to the high social costs. Some studies, however, have called for more theoretical nuance, pointing toward variation in how norms are perceived (Sulaiman, Kipchumba, & Magan, 2017) and the degree to which people adhere to the local social convention (Efferson, Vogt, Elhadi, Ahmed, & Fehr, 2015). Most significantly, application of the theory of FGC as a social convention to understand and promote change relies on the initiatives of a substantial proportion of the population to oppose and abandon the existing social convention upholding FGC. It thus relies on divergence and opposition to the norm.

This divergence leads to a need to pay more attention to the conceptualization of community and social norm. I think it is time to challenge the conceptualization of a community as a clear and defined entity with a uniform social convention. In a contemporary context, individuals and families encounter multiple communities and networks with diverging social norms. Furthermore, migration has led to changes in the constitution and power balance of decision-making groups (Johansen, 2019). Thus, the addition of personal attitude and perception of power into a model of understanding FGC seems particularly pertinent in diaspora (Brady et al., 2019; Montano & Kasprzyk, 2015; Ziyada et al., in press). In the following section, I will outline some important nuances to consider in linking the theory of planned behavior to research and provision of care.

## Nuancing Major Elements

A major strength of the theory of planned behavior is the simplicity of the model and its applicability in assessing major factors affecting FGC decision making. In reality, however, it can be challenging to clearly delineate these factors, as they are deeply interconnected. For example, it is hard to imagine a person holding a positive attitude to FGC and a negative attitude to deinfibulation unless the person has been raised in a community in which infibulation constitutes a social norm. Likewise, it is hard to assess perceived control without taking into account the perceived social norms and, thus, fear of normative repercussions. Consequently, while the distinction of major factors affecting FGC decision making can be a useful tool facilitating reflection, in reality these factors are interconnected.

## Nuancing Attitudes

In their model, Brady et al. (2019) include not only positive or negative attitudes to deinfibulation, but also attitude to FGC in general, infibulation, as well as timing of deinfibulation, its extent, and maintenance. This is important as it takes into account that the attitudes to these various aspects of the practice might diverge, which complicates the issue. Among these factors, I would suggest we focus most on the timing of deinfibulation, as this takes into account the changing cultural meaning of FGC over a woman’s life span. Premarital deinfibulation is highly controversial as it undermines the major cultural meaning of infibulation: the creation, protection, and evidence of virginity and virtue (Johansen, 2017a; Ziyada et al., in press). Deinfibulation conducted at marriage or childbirth, in contrast, has positive connotations relating to marriage and motherhood. However, the method and extent of deinfibulation can be perceived as controversial, as they can challenge other cultural values. In particular, both can challenge ideals of virility at marriage, as well as perceptions of the necessity of a tight vaginal introitus, safeguarded by some degree of infibulation, which is seen as fundamental to ensuring male sexual satisfaction and, thus, marital stability (Johansen, 2017b, c; Ziyada et al., in press).

These factors are major reasons for the widespread resistance to deinfibulation, even among women who hold a negative attitude about infibulation. Thus, whereas World Health Organization’s recent guidelines on the management of infibulation suggest that such surgeries should be offered (WHO, 2018), women reported that they want a stronger recommendation, underpinned by solid insight into the benefits of such a procedure (Ziyada et al., in press). Such knowledge could, thus, support women in making choices that favor health benefits over sociocultural expectations.

Another strength of Brady et al.’s (2019) model is that it includes questions on sense of belonging and identity in the assessment of attitude. This points to the importance of identifying major referents and, as such, opens up the possibility that women may relate to various social networks and cultural values. Several recent studies indicate how migrant women maneuver between a host of social networks, for which they hold a variety of perceived social norms (Johansen & Ahmed, 2020). The relative significance of various networks seems to vary partly with life stage. For example, young unmarried women were most scared of conducting premarital deinfibulation when they did not know their future husband, where he was raised, and thus what values he, and his family, would have regarding infibulation, deinfibulation, and virginity (Johansen, 2017a). In a study by Johansen (2019), young Somali women reported an expectation of clan endogamy. Thus, while they did not think that Somali men raised in Norway would expect an infibulated wife, they were less sure about men raised elsewhere. This meant that some women resisted deinfibulation in spite of support from all significant others, for fear of the expectation that they would have to find an unknown future husband in another country. However, a minority of women were adamant that they would marry outside their community, and most of these saw premarital deinfibulation as an opportunity to escape health problems and appear as a woman without FGC (Johansen & Ahmed, 2020; Ziyada et al., in press).

Deinfibulation at marriage and childbirth is a physical necessity and, thus, less controversial. However, while it does not challenge the value of virginity, it challenges values related to virility and male sexual pleasure (Johansen, 2017a, b, c, 2019). Thus, a recent study found that while traditional forms of deinfibulation involve significantly more pain over a longer period of time, most Somali and Sudanese research participants had resorted to traditional means of deinfibulation. In this context, the controversy concerns mainly the form of deinfibulation (i.e., medicalization) and, to some extent, the extent of deinfibulation (Johansen, 2017b, c).

I further want to highlight two additional factors that contribute to resistance against deinfibulation not mentioned in Brady et al.’s (2019) Target Article, namely fear of pain and retraumatization. Almost all women I have interviewed over the years recall their infibulation as an extremely painful and terrifying experience (Johansen, 2002) and fear that deinfibulation would be a similar experience. However, most women reported that they rarely shared fear or eventual experiences of flashback and retraumatization with the provider.

Overall, then, I suggest that when applying the model formulated by Brady et al. (2019), it could be useful to add a stronger focus on some of the underlying elements, namely sexuality and fear of the procedure itself. I consider exploration of women’s perceptions of female sexuality and how these may be interlinked with perceptions of infibulation and deinfibulation, as necessary to employ Brady et al.’s model. In cases where women are in stable relationships, one should further explore whether it could be beneficial to involve the partner in discussions. Recent studies have found partners to be the main referent in deliberations about deinfibulation at marriage and childbirth (Ziyada et al., in press).

## Nuancing Perceived Norms

Brady et al.’s (2019) treatment of perceived norms is useful in that it focuses on how norms are *perceived*, rather than talking about social norms as objective facts (as it could be the case with the theory of FGC as a social convention). This allows for a more flexible and nuanced understanding of the interplay between personal and social factors. Several studies have found that different individuals within the same community may have different perceptions of social norms and these perceptions may differ from what can be assessed as common social norms. One example, a recent study of Somali migrants, of whom most lived in a small Norwegian town, found that while the majority of participants expressed a positive attitude to deinfibulation personally, they all perceived it as totally unacceptable in their community (Johansen, 2019).

Furthermore, it is likely that norms and expectations expressed from various referents may be different and contradictory given the current trends of change. Women in diaspora may maneuver between a conglomerate of different perceived norms (Johansen, 2017a, b, 2018; Ziyada et al., in press). This can include those of close family and relatives in countries of origin, ethnic/clan, and national networks in country of residence and transnationally, as well the perceived norms of other social groups they relate to, including that of their host community. Which social norms women in diaspora relate to the most when making decisions regarding FGC may thus vary. For example, a woman may perceive the social norms of her own ethnic community in diaspora and at home as condemning deinfibulation. However, she may still choose to disregard this when making her decision and instead consider the social norms of the host community (Johansen & Ahmed, 2020; Ziyada et al., in press).

The potential gap between “actual” and “perceived” social norms may be even larger in the context of exile, not only because of the multiplicity of social norms, but also because FGC has become increasingly privatized and silenced (Johansen, 2019; Lunde, Sagbakken, & Johansen, 2019). Thus, whereas the social norms regarding FGC traditionally were expressed in a myriad of ways, they have become overwhelming silent among Somali migrants in Norway. It may thus be more difficult to assess whether there is a common social norm and what it is. Furthermore, are perceived norms intimately linked to perceived control, as indicated above? Women’s’ willingness and ability to act contrary to the perceived norms of one’s family and/or ethnic community are linked to relative power as well as willingness to pay the price of negative reactions from one’s community.

## Nuancing Perceived Control

In their choice of the theory of planned behavior for exploration of FGC, Brady et al. (2019) emphasized the usefulness of including perceived control in the model. They outlined perceived control mainly in relation to autonomy over the medical decision-making process itself and in relation to the wishes of significant others, such as partner, family, or community. However, I think it would be useful to link the concept of perceived control more strongly to perceptions of social norms, at least in the context of diaspora. That is, the question as to whether the woman feels able to make an autonomous decision needs to be combined with fear of repercussions from breaking perceived norms.

Prior to their first marriage, Somali and Sudanese migrant women express a sense of limited autonomy. Even though women above the age of 16 can legally seek medical care without parental consent, many young women reported that they could not do it, as they feared their mothers would find out, most commonly due to changes in their urinary pattern (i.e., more noisy urination). This again could cause suspicion of premarital sexual engagement. Some young women whose mothers supported or even encouraged deinfibulation still resisted, for fear of disapproval from a future husband. Furthermore, almost all young women claimed that if they were to return to Somalia, having undergone premarital deinfibulation would cause social exclusion and condemnation (Johansen, 2017a, b, 2018, 2019; Ziyada et al., in press). Thus, perceived control also relates to perceived social sanctions. This is again related to plans and hopes for the future, including where one plans to live, who one hopes to marry. After marriage, women tend to consider their husband as the main referent, and his views would strongly influence the likelihood of whether or not to seek deinfibulation (Johansen, 2017a, b, c; Ziyada et al., in press). Some women, however, resisted deinfibulation even in cases where the husband encouraged it (Johansen, 2017b).

Hence, perceived control in a sense also measures vulnerability *vis*-*à*-*vis* various significant others, which varies between individuals and groups. Several recent studies have identified some systematic distinctions in this regard (Johansen & Ahmed, 2020; Ziyada et al., in press). A set of various factors, including age/seniority, education, and employment, were systematically associated with perceived control. That is, young women and women with little, or no, education and a weak position in the work market tended to identify more strongly with their own ethnic group/clan or nationality and tended to act according to the perceived norms of their ethnic group. In contrast, some of the more mature women, particularly women with higher education and good employment, expressed a more critical stand toward the perceived norms of their ethnic community and tended to make more autonomous decisions. To the extent that their decisions were influenced by social norms, these were more commonly the social norms associated with modern, Western life. They were fully aware that their choices contradicted major traditional values, but were willing to accept the social costs. For some, these choices included marrying outside their ethnic community, moving to areas with few other migrants, and mainly socialization with likeminded women (Johansen & Ahmed, 2020). They rarely severed the bonds to their own ethnic communities, but redefined them, often by engaging in volunteer community work.

However, the concept of perceived control as described in the model could be very useful in exploring decision making regarding whether or not to perform FGC itself. As FGC decision making traditionally involves several different persons, this interdependency needs to be further explored particularly in contemporary society, in which both the composition and power balance within the decision-making group are changing (Bjalkander, Leigh, Harman, Bergstrom, & Almroth, 2012; Eldin, Babiker, Sabahelzain, & Eltayeb, 2018; Hernlund & Shell-Duncan, 2007; Sabahelzain, Eldin, Babiker, Kabiru, & Eltayeb, 2019).

## Conclusion

Overall, I find that Brady et al.’s (2019) Target Article provides a promising new conceptualization for analyzing FGC decision making. While the Target Article was developed to explore a specific form of health care for a particular form of FGC, the model has a much broader usage. The theory of planned behavior has formerly been used to assess intention to perform, or not perform, FGC in countries of origin (Ilo et al., 2018; Pashaei, Ponnet, Moeeni, Khazaee-pool, & Majlessi, 2016) and can most likely be employed to assess intention for other forms of health care after FGC in both countries of origin and migration.

However, its usage needs to be built on broader insights into the nuances and complexities of current FGC discourses in various communities, as suggested in this Commentary. This includes a stronger emphasis on sexual concerns, which are at the root of much FGC decision making, particularly regarding infibulation and deinfibulation (Berg & Denison, 2013a, b; Johansen, 2007, 2017a, b, c; Ziyada et al., in press).

Another major aspect is time. That is, many women may need time to reflect between repeated consultations before making a decision of whether or not to undergo deinfibulation. This might specifically be the case for women in stable relationships, where there is also a need to consider including their partner in the deliberations (Johansen, 2017a, b; Johnson-Agbakwu, Helm, Killawi, & Padela, 2014; Ziyada et al., in press).

Another aspect that could be added to strengthen the model is the intensity of attitude. In our studies, we sensed a connection between intensity of conviction, intention, and behavior. For example, many who feel negatively about FGC expressed a willingness to accept so-called sunna circumcision as a compromise. In most communities practicing FGC, a major element in the discourse of change is a tendency to argue for change in the type of FGC performed, rather than full abandonment. Sunna circumcision is a common term for this alterative cut, often perceived as less extensive and thus less harmful than infibulation (Johansen, 2019). There is, however, no common agreement on the anatomical extent of what is referred to as sunna circumcision, and it can describe anything from a minor prick to a slightly less extensive infibulation (Elmusharaf, Elhadi, & Almroth, 2006; Johansen, 2019; Powell & Yussuf, 2018). On the other hand, some women have taken serious actions, such as migration and going underground, to be able to act according to their convictions and the same in the case of men, who have been found to be a decisive factor in abandonment of FGC (Johansen, 2019; Johansen & Ahmed, 2020).

A final aspect of FGC that has not been touched neither in Brady et al.’s (2019) Target Article or my Commentary is that, in some communities, infibulation is valued also for its aesthetic appearance and thus deinfibulation is considered to render female genitalia as ugly and unattractive. Given the rise of genital cosmetic surgeries, particularly in Western countries, dealing with such concerns would require further considerations that is beyond what I can explore in this Commentary.
